# RecurIndex assay as an aid for adjuvant chemotherapy decisions in HR-positive HER2-negative breast cancer patients

**DOI:** 10.3389/fonc.2022.896431

**Published:** 2022-12-07

**Authors:** Haibo Wang, Li Ma, Yanan Zhang, Ouchen Wang, Zhimin Wei, Xiaohong Xie, Xiaoming Zha, Jian Zeng, Qing Lv, Yu Ren, Huimin Wang, Furong Du, Shangzhi Cao

**Affiliations:** ^1^ Department of Breast Center, The Affiliated Hospital of Qingdao University, Qingdao, China; ^2^ Department of Breast Center, The Fourth Hospital of Hebei Medical University, Shijiazhuang, China; ^3^ Breast Disease Center, Zhongda Hospital, School of Medicine, Southeast University, Nanjing, China; ^4^ Department of Breast Surgery, The First Affiliated Hospital of Wenzhou Medical University, Wenzhou, China; ^5^ Department of Pathology, The Affiliated Hospital of Qingdao University, Qingdao, China; ^6^ Department of Breast Surgery, The First Affiliated Hospital of Zhejiang Chinese Medical University, Hangzhou, China; ^7^ Breast Disease Department, The First Affiliated Hospital of Nanjing Medical University, Nanjing, China; ^8^ Department of Gastrointestinal and Gland Surgery, The First Affiliated Hospital of Guangxi Medical University, Nanning, China; ^9^ Department of Breast Surgery, West China Hospital, Sichuan University, Chengdu, China; ^10^ Department of Breast Surgery, The First Affiliated Hospital of Xi’an Jiaotong University, Xi’an, China; ^11^ State Key Laboratory of Translational Medicine and Innovative Drug Development, Jiangsu Simcere Diagnostics Co., Ltd., Nanjing, China

**Keywords:** Early breast cancer, Luminal type, RecurIndex assay, Adjuvant chemotherapy, Survival outcomes

## Abstract

**Background:**

Adjuvant chemotherapy is a major adjuvant treatment modality for hormonal receptor (HR)-positive and HER2-negative early breast cancer, but only 2%-20% of patients derive practical benefits. How to balance its potential benefits and risks becomes a challenging clinical problem. The purpose of this study was to assess whether RecurIndex assay could serve as an aid for adjuvant chemotherapy decisions in Chinese patients with HR-positive HER2-negative early breast cancer.

**Methods:**

The tissue samples of pT1-2N0 HR-positive HER2-negative breast cancer from multiple centers were detected using RecurIndex assay, based on which the patients were assigned into low- and high-risk groups. The survival outcomes of low- and high-risk patients including those with and without adjuvant chemotherapy were compared, and the risk factors for recurrence and metastasis were identified.

**Results:**

Totally 445 patients were eligible for analysis. By contrast to high-risk patients, low-risk patients represented better 7-year recurrence-free survival (RFS), distant recurrence-free survival (DRFS) and local recurrence-free survival (LRFS) rates. For low-risk patients, no significant differences were shown between those with and without adjuvant chemotherapy in 7-year RFS, DRFS and LRFS rates. These differences were also inapparent between high-risk patients with and without adjuvant chemotherapy. The multivariate model revealed high-risk patients had a significantly elevated risk of recurrence and metastasis than those at low risk.

**Conclusion:**

HR-positive HER2-negative early breast cancer patients at low risk stratified by RecurIndex assay might be exempt from adjuvant chemotherapy. Whether adjuvant chemotherapy may derive survival benefits for high-risk patients still needs larger cohorts to verify.

## Introduction

Breast cancer is the most common malignancy in women, ranking the second among the causes for cancer-associated deaths in women (1). The clinical outcomes of breast cancer, a molecularly heterogenous disease, depend on its biological subtypes, which is related to diverse molecular characteristics (2, 3). Several markers including estrogen receptors (ER), progesterone receptors (PR), human epidermal growth factor receptor 2 (HER2) and Ki67 have been demonstrated to play crucial roles in the treatment of early breast cancer (EBC) (4). It is estimated that luminal type accounts for 70% of all breast cancers and approximately 85% of EBCs (5–7). The prognosis of patients with luminal EBC is usually good, having a 5-year disease-free survival (DFS) rate of 99.1% for T1N0 luminal breast cancer (8). Although with good overall prognosis, up to 5% of patients still incur recurrence (7). Application of adjuvant chemotherapy in EBC is thought as an alternative therapy for patients with operable breast cancer. However, only a small minority of patients derive benefits, and others may at risk of toxicities (9). How to balance the potential benefits and risks of adjuvant chemotherapy in EBC becomes a challenging clinical problem.

With emerging multigene assays, breast cancer treatment is changing. Currently, National Comprehensive Cancer Network (NCCN) guidelines for breast cancer propose that multigene assays comprising 21-gene recurrence score (RS) assay (Oncotype Dx) and 70-gene signature (MammaPrint) are conductive to guiding the use of adjuvant chemotherapy in some patients with hormonal receptor (HR)-positive and HER2-negative breast cancer (10). However, these multigene assays are developed based on the western population, and whether they are appropriate to the Asian population needs further analysis.

RecurIndex, a risk assessment model including diverse clinicopathological characteristics and genes closely related to breast cancer, is developed based on the Chinese population and has been demonstrated to be capable of predicting the long-term distant recurrence-free interval (DRFI) and recurrence-free survival (RFS) in patients with primary operable breast cancer, especially those with N0, ER/PR-positive and HER2-negative patients (11, 12). Recently, a study on the application of RecurIndex assay in pT1-2N1M0 breast cancer has revealed that post-mastectomy radiotherapy (PMRT) may be avoided for low-risk patients assessed by RecurIndex assay but beneficial for high-risk patients (13). Nevertheless, there are few studies on the association of RecurIndex assay with adjuvant chemotherapy in EBC. In this study, we leveraged RecurIndex assay to stratify Chinese patients with HR-positive HER2-negative EBC as high and low groups and compared their survival outcomes according to the presence or absence of adjuvant chemotherapy, thus guiding the clinical decision-making on the use of adjuvant chemotherapy.

## Materials and methods

### Study population

Patients with pT1-2N0 HR-positive HER2-negative breast cancer who received surgery in 9 medical centers between 2013 and 2016 were included in this retrospective study. Their formalin-fixed and paraffin-embedded (FFPE) tumor samples were obtained for RecurIndex assay. The inclusion criteria included: (1) women aged 18-75 years; (2) pT1-2N0 HR-positive HER2-negative breast cancer diagnosed by both immunohistochemistry (IHC) staining and RecurIndex assay; (3) breast-conserving surgery or mastectomy as the initial treatment; (4) sufficient FFPE tumor samples, complete clinicopathological and follow-up data. The patients would be excluded from the study if they met any one of the criteria below: (1) preoperative administration of neoadjuvant therapy including chemotherapy; (2) incomplete postoperative endocrine therapy and/or chemotherapy; (3) patients diagnosed as bilateral breast cancer, or with the history of other malignancies.

The study was performed according to the principles of Declaration of Helsinki and local regulations. All the patients were informed consent, and the study was approved by the Institutional Review Board of The Affiliated Hospital of Qingdao University (approval No.: QYFYKYLL801311920).

### RecurIndex assay

The genes included in the RecurIndex assay was composed of 18 genes (*TRPV6*, *DDX39*, *BUB1B*, *CCR1*, *BLM*, *C16ORF7*, *PIM1*, *TPX2*, *PTI1*, *TCF3*, *NFATC2IP*, *OBSL1*, *MMP15*, *ESR1*, *ERBB2*, *CLCA2*, *SF3B5*, and *PHACTR2*), along with 3 housekeeping genes (*ACTB*, *RPLP0* and *TFRC*). All these genes were simultaneously measured in diverse wells. Specifically, the primer pairs of the target genes were placed into PanelChip^®^, and then the total ribonucleic acid (RNA) extracted from FFPE tumor tissues was used to perform reverse-transcriptase (RT) quantitative polymerase chain reaction (qPCR) on the PanelStation platform (Quark BioSciences, Inc., Hsinchu, Taiwan). The expression of each gene in the FFPE samples was measured using qPCR. Normalization of gene expression was computed as delta CT = 25 - CT (gene of interest) + CT (mean of housekeeping genes). The RNeasy FFPE Mini Kit (Qiagen, Valencia, CA, USA) was used to extract RNA from the FFPE tissue sections with 5-10 μm in thickness, and Qubit 4.0 fluorometer (Thermo Fisher Scientific, Massachusetts, USA) and NanoDrop Microvolume Spectrophotometer (Thermo Fisher Scientific, Massachusetts, USA) were employed for determining RNA concentration. Subsequently, the extracted RNA was reserved at -80°C until use. Through the RT^2^ First Strand and RT^2^ SYBR Green ROX qPCR MM kits (Qiagen, Valencia, CA, USA), a total of 800 ng RNA was utilized for RT-qPCR. Briefly, 2X SYBR MasterMix, 60×ROX, 100× qPCR Control diluent, Nuclease-Free Water, and cDNA products were used for preparing the qPCR system. The sample loader attached to the PanelStation was selected, then 350 μL heat conductive oil and 80 μL qPCR mixture were loaded to the PanelChip^®^ and the chip was loaded to PanelStation. Finally, thermal cycling was performed at 95°C for 44 seconds and 60°C for 88 seconds, with 40 cycles in total.

As a previous study described (11), the genetic and clinical factors were combined based on the Cox proportional hazard model to determine their weighting, and concordance statistics (C index) with the receiver operating characteristic curve were used to determine the most effective model. The best cutoff values for predicting local recurrence and distant recurrence were identified by Logistic regression.

### Extraction of clinicopathological and follow-up data

Through reviewing the electronic medical record of patients, the clinicopathological information was extracted, containing the age, tumor stage, histological grade, presence or absence of local recurrence, distant recurrence and lymphovascular invasion (LVI), as well as the conditions of adjuvant chemotherapy and adjutant endocrine therapy. All the patients were followed up after surgery through telephones and further consultations.

The RFS rate was regarded as the primary study endpoint, and distant recurrence-free survival (DRFS) and local-regional recurrence-free survival (LRFS) rates were as the secondary endpoints. RFS was defined as the duration of time from surgery until any recurrence of ipsilateral chest, breast, regional lymph node recurrence, distant metastases, or death occurred from any cause. DRFS was defined as the duration of time from surgery until distant recurrence, or death occurred from any cause. LRFS was defined as the duration of time from surgery until any recurrence of ipsilateral chest, breast, regional lymph node recurrence, or death occurred from any cause.

### Identification of HR-positive HER2-negative breast cancer

HR and/or HER2 status was assessed based on the guidelines (14). ER/PR was considered positive when there were at least 1% positive tumor nuclei by IHC assay. Patients with grade I-II, ER/PR-positive and HER2-negative breast cancer were classified into IHC luminal A subtype, whereas those with grade III, ER/PR-positive and HER2-negative breast cancer were categorized into IHC luminal B subtype (15, 16).

### Statistical analysis

In this study, *t* test was used for comparison of continuous variables with normal distribution, manifesting as the mean (standard deviation, SD); Mann-Whitney U rank sum test was employed to compare continuous variables with abnormal distribution, describing as medians with interquartile range. Categorical variables expressing as frequencies and percentages were compared using Fisher’s exact test or χ^2^ test. Survival probabilities were estimated utilizing the Kaplan-Meier method and compared using Log-rank test. The multivariate Cox proportional-hazards model was performed to analyze the influencing factors for recurrence and metastasis. All statistical tests were two-tailed, with statistically significant differences at *p* values <0.05. Statistical analyses were performed using SPSS 24.0 statistical software (SPSS Inc., Chicago, IL, USA).

## Results

### Baseline characteristics of study population

In this multi-center study, totally 610 patients were diagnosed as pT1-2N0 HR-positive HER2-negative breast cancer between 2013 and 2016. When 13 cases aged <18 or >75 years, 87 cases lacking follow-up data, 61 cases failing in RecurIndex assay (the proportion of tumor cells ≤30%), 3 cases receiving neoadjuvant chemotherapy and 1 with history of other malignancies were excluded, 445 eligible cases remained for analysis. Their median follow-up duration was 6.25 years.

There were 344 low-risk patients and 101 high-risk patients according to RecurIndex assay. It can be found that statistically significant differences were all shown between low- and high-risk patients, including age (*p*<0.001), tumor stage (*p*<0.001), histological grade (*p*<0.001), LVI (*p*<0.001), local recurrence (*p*=0.001), distant recurrence (*p*=0.002), adjuvant chemotherapy (*p*<0.001) and endocrine therapy (*p*<0.049) (**Table 1**). Through IHC assay, 347 cases were assessed as Luminal A subtype, while 98 cases as Luminal B subtype. Except for the age (*p*=0.082), the differences were all pronounced between luminal A- and B-type patients regarding all other characteristics (*p*<0.05; **Table S1**).

**Table 1:** Baseline characteristics of 445 patients with pT1-2N0 HR-positive HER2-negative breast cancer, n(%).

Characteristics	RecurIndex assay		*p*
	Low risk (*n*=344)	High risk (*n*=101)	
Median age (range), years	51(29-82)	46 (26-67)	<0.001
Tumor stage			<0.001
T1	270 (78.5)	39 (38.6)	
T2	74 (21.5)	62 (61.4)	
Histological grade			<0.001
I	44 (12.8)	4 (4.0)	
II	261 (75.9)	38 (37.6)	
III	39 (11.3)	59 (58.4)	
Lymphovascular invasion			<0.001
Yes	34 (9.9)	38 (37.6)	
No	310 (90.1)	63 (62.4)	
Local recurrence			0.001
Yes	5 (1.5)	9 (8.9)	
No	339 (98.5)	92 (91.1)	
Distant recurrence			0.002
Yes	15 (4.4)	13(12.9)	
No	329 (95.6)	88 (87.1)	
Adjuvant chemotherapy			<0.001
TC	103 (29.9)	33 (32.7)	
Anthracycline without taxane	28 (8.1)	17 (16.8)	
Anthracycline plus taxane	19 (5.5)	26 (25.7)	
CEF	12 (3.5)	15 (14.9)	
Others	12 (3.5)	6 (5.9)	
No chemotherapy	170 (49.4)	4 (4.0)	
Adjuvant endocrine therapy			<0.049
Tam/TOR	178 (51.7)	54 (53.5)	
AI	124 (36.0)	29 (28.7)	
OFS+Tam/TOR	2 (0.6)	4 (4.0)	
OFS+AI	3 (0.9)	0 (0.0)	
Others	15 (4.4)	10 (9.9)	
None reported	8 (2.3)	1 (1.0)	
No endocrine therapy	14 (4.1)	3 (3.0)	

TC, taxane and cyclophosphamide; CEF, cyclophosphamide/epirubicin/fluorouracil; OFS, ovarian function suppression; Tam, tamoxifen; TOR, toremifene; AI, aromatase inhibitor.

### Association of RecurIndex and IHC assays with survival outcomes

**Figure 1:**
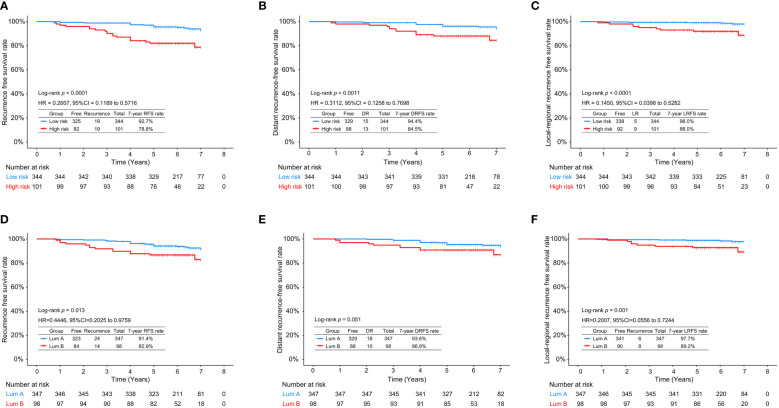
The 7-year recurrence-free survival rate **(A)**, distant recurrence-free survival rate **(B)**, and local recurrence-free survival rate **(C)** in low- and high-risk patients stratified by RecurIndex assay; The 7-year recurrence-free survival rate **(D)**, distant recurrence-free survival rate **(E)**, and local recurrence-free survival rate **(F)** in luminal A-subtype and luminal B-subtype patients stratified by immunohistochemistry staining.

**Figure 1** shows the 7-year RFS, DRFS and LRFS rates of the low- and high-risk patients stratified by RecurIndex assay and IHC assay. It can be observed that the low-risk patients stratified by RecurIndex assay had higher 7-year RFS rate (92.7% *vs*. 78.8%, *p*<0.0001; **Figure 1A**), DRFS rate (94.4% *vs.* 84.5%, *p*=0.0011; **Figure 1B**) and LRFS rate (98.0% *vs.* 88.0%, *p*<0.0001; **Figure 1C**) compared with the high-risk patients. According to the results of IHC assay, the 7-year RFS rate (91.4% *vs.* 82.9%, *p*=0.013; **Figure 1D**) and LRFS rate **(**97.7% *vs.* 89.2%, *p*=0.001; **Figure 1F** **)** in patients with luminal A subtype were both superior to those in patients with luminal B subtype, but the difference was indistinctive in the 7-year DRFS rate (93.6% *vs.* 86.9%, *p*=0.051; **Figure 1E**).

**Figure 2:**
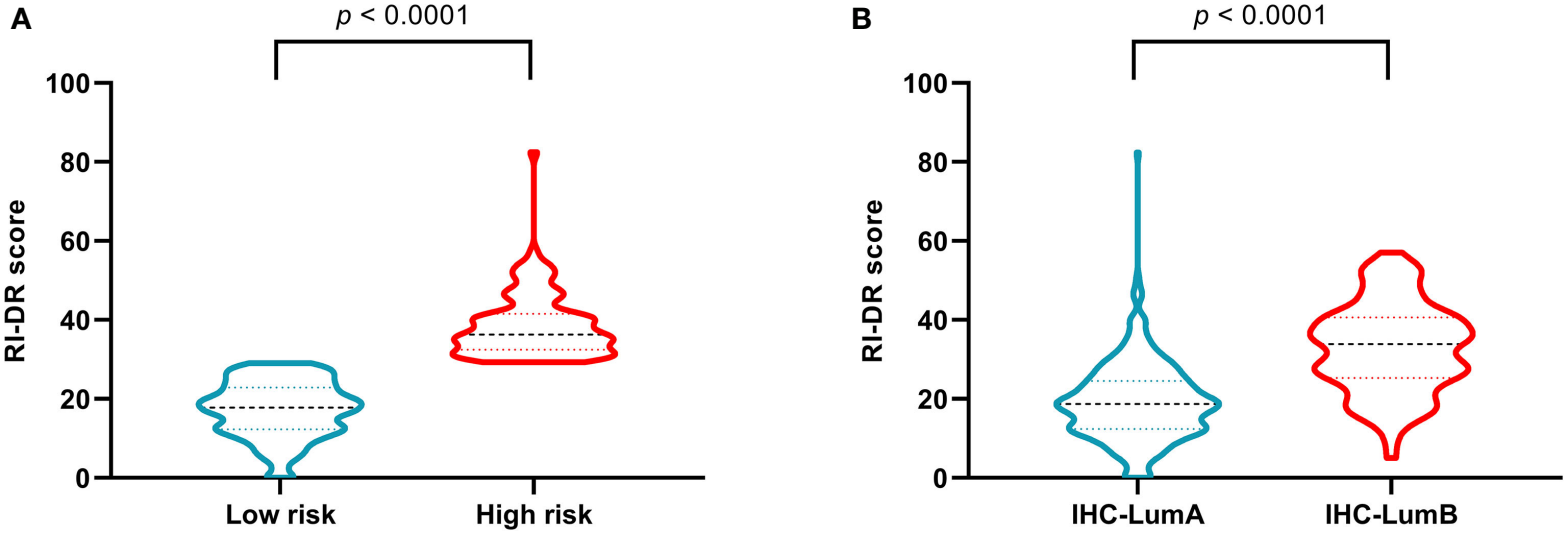
Recurrence index-distant recurrence scores in patients stratified by RecurIndex assay **(A)** and by immunohistochemistry staining **(B)**.

The recurrence index for distant recurrence (RI-DR) scores of the high-risk patients assessed by RecurIndex assay were significantly higher than those of the low-risk patients (*p*<0.0001; **Figure 2A**). Similarly, patients with luminal B subtype had significantly increased RI-DR scores than those with luminal A subtype (*p*<0.0001; **Figure 2B**).

### Subgroup analyses of survival outcomes

**Figure 3:**
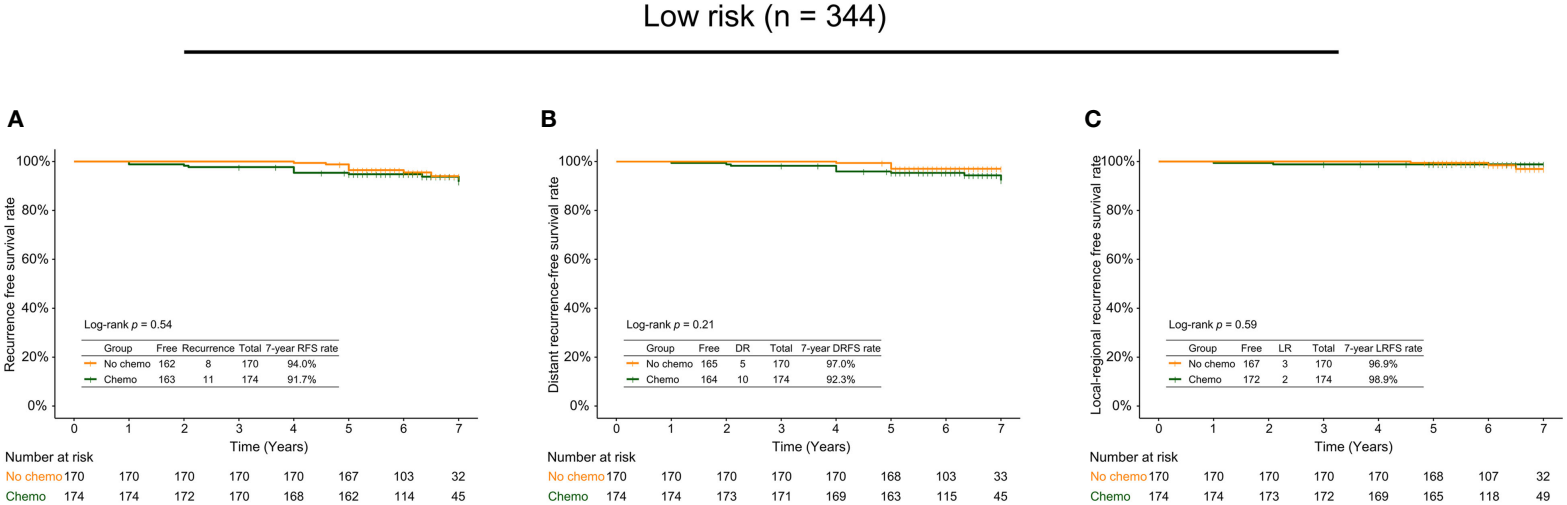
The 7-year recurrence-free survival rate **(A)**, distant recurrence-free survival rate **(B)**, and local recurrence-free survival rate **(C)** in low-risk patients with and without adjuvant chemotherapy.

Of 344 low-risk patients, 174 cases received adjuvant chemotherapy, while 170 didn’t. The subgroup analysis based on chemotherapy indicated no statistically significant differences between the low-risk patients with and without adjuvant chemotherapy regarding the 7-year RFS (94.0% *vs*. 91.7%, *p*=0.54; **Figure 3A** **)**, DRFS (97.0% *vs*. 92.3%, *p*=0.21; **Figure 3B**), and LRFS (96.9% *vs*. 98.9%, *p*=0.59; **Figure 3C** **)** rates. Of 101 high-risk patients, there were 97 cases with adjuvant chemotherapy and 4 without adjuvant chemotherapy. The 7-year RFS and DRFS rates of the high-risk patients with adjuvant chemotherapy were relatively higher than those without, but without significant differences (RFS rate: 78.8% *vs*. 75.0%, *p*=0.70; DRFS rate: 84.7% *vs*. 75.0%, *p*=0.43).

To further assess the benefits of adjuvant chemotherapy, we used an approximate menopausal status defined by an age cutoff at 50 years to classify the low-risk patients into the group aged over 50 years and the group aged 50 years or younger. It could be observed that there were no significant differences in survival outcomes whether in the group aged 50 years or younger (RFS rate: 90.5% *vs*. 90.7%, *p*=0.95, **Figure S1A**; DRFS rate: 96.6% *vs*. 90.7%, *p*=0.34, **Figure S1B**) or the group aged over 50 years (RFS rate: 95.9% *vs*. 93.3%, *p*=0.57, **Figure S1C**; DRFS rate: 97.3% *vs*. 94.7%, *p*=0.63, **Figure S1D**). Notably, 330 out of 344 low-risk patients received endocrine therapy. Based on presence or absence of adjuvant chemotherapy, 330 patients were further divided into two groups. The results showed that the differences were inapparent between the patients receiving endocrine therapy with/without chemotherapy regarding RFS (94.3% *vs*. 91.5%, *p*=0.41, **Figure S1E**) and DRFS (97.5% *vs*. 92.1%, *p*=0.13, **Figure S1F**) rates.

### Risk factors for recurrence and metastasis

The risk factors for recurrence and metastasis in all patients were analyzed using a multivariate Cox proportional-hazards model. As shown in **Table 2**, the risk of recurrence and metastasis was significantly higher in high-risk patients than in low-risk patients stratified by RecurIndex assay (hazard ratios [HR]: 3.106, 95% confidence interval [CI]: 1.598-6.035, *p*=0.001); patients with LVI had a higher risk of recurrence and metastasis compared with those without (HR: 2.730, 95%CI: 1.348-5.528, *p*=0.005).

**Table 2:** Uni- and multi-variate analyses of recurrence and metastasis in all patients (n=445).

Variables		Univariate		Multivariate	
		HR (95% CI)	*p*	HR (95% CI)	*p*
Age, years	<40	Reference		Reference	
	≥40	0.574 (0.252-1.304)	0.185	0.769 (0.328-1.803)	0.546
Tumor stage	T1	Reference		Reference	
	T2	2.367 (1.253-4.472)	0.008	1.533 (0.748-3.143)	0.243
Histological grade	I	Reference		Reference	
	II	1.775 (0.417-7.550)	0.437	1.651 (0.375-7.258)	0.507
	III	3.753 (0.853-16.517)	0.080	2.014 (0.417-9.729)	0.384
Sample size from different centers	<50	Reference		Reference	
	50-150	1.041 (0.439-2.471)	0.927	0.996 (0.407-2.441)	0.994
	150-300	0.840 (0.401-1.763)	0.646	1.225 (0.555-2.701)	0.615
RecurIndex assay	Low risk	Reference		Reference	
	High risk	3.845 (2.034-7.267)	<0.001	3.106 (1.598-6.035)	0.001
Lymphovascular invasion	No	Reference		Reference	
	Yes	3.518 (1.818-6.808)	<0.001	2.730 (1.348-5.528)	0.005
Adjuvant chemotherapy	No	Reference		Reference	
	Yes	2.146 (1.015-4.534)	0.046	1.040 (0.425-2.547)	0.931
Adjuvant endocrine therapy	No	Reference		Reference	
	Yes	1.443 (0.198-10.526)	0.718	1.886 (0.243-14.630)	0.544

HR, hazards ratios; CI, confidence interval.

## Discussion

Currently, adjuvant chemotherapy plays a crucial role in the adjuvant treatment of HR-positive and HER2-negative EBC, while only 2%-20% of patients derive practical benefits, others may endure long-term treatment-associated toxicities (17), emphasizing the importance of effective and well-stratified patients in therapy. Although multigene assays developed in the western population have informed risk-stratification by assessing the postoperative recurrence risk based on gene-expression profiling, there are certain ethnic differences for the Asian population. South Asian women with stage I breast cancer were found to have the lowest risk of death at 7 years compared with non-Hispanic and black women, which may be explained by intrinsic biological differences including distant metastasis and lymph node metastasis (18). Recently, a next-generation sequencing-based multigene assay has been developed to predict the risk of distant recurrence in ER-positive, HER2-negative breast cancer from the Korea population (19). Differently, RecurIndex assay is developed based on RT-qPCR, and combines the gene-expression profiling and clinicopathological features of Chinese breast cancer patients to analyze the risk of local recurrence and distance recurrence. The present study first demonstrated the clinical utility of RecurIndex assay for guiding adjuvant chemotherapy decisions in Chinese HR-positive HER2-negative EBC patients.

The present study totally enrolled 344 low- and 101 high-risk patients stratified by RecurIndex assay. Statistically significant differences were shown between the low- and high-risk patients regarding age, tumor stage, histological grade and LVI, highlighting the correlation between RecurIndex assay and these clinicopathological features previously confirmed to be able to assess the risk of breast cancer (20–22). Moreover, low-risk patients harbored a decreased risk of recurrence and metastasis and better 7-year RFS, DRFS and LRFS rates compared with high-risk patients, suggesting the accuracy of RecurIndex assay in risk stratification of patients and its importance as a promising complementary tool to be predictive of prognosis, in agreement with recent findings (11, 23, 24). Of note, adjuvant chemotherapy did not improve the survival outcomes of low-risk patients, offering clinicians an opportunity to identify the patients at low risk for whom chemotherapy can be omitted to avoid treatment-related toxicities.

There is uncertainty regarding the benefit of chemotherapy in EBC, although various multigene assays are available. It was estimated about 46% of EBC women at high clinical risk might not need chemotherapy (25). The subsequent study showed the distant metastasis-free survival rate of patients at low clinical and low genomic risk assessed by 70-gene signature who only received endocrine therapy for 8 years was optimal, suggesting this group of patients might not need to use chemotherapy (26). Moreover, the low-risk patients with HR-positive and HER2-negative breast cancer who were stratified by a 21-gene assay were also recommended to use endocrine therapy alone, with no need for adjuvant chemotherapy (27), supporting our results that low-risk patients stratified by RecurIndex assay could omit adjuvant chemotherapy to avoid overtreatment. In women with ER-positive and lymph node-negative breast cancer using 21-gene assay for risk stratification, the patients with high-RS tumors derived a significant benefit from chemotherapy, those with low-RS tumors showed a minimal benefit, while those with intermediate-RS tumors seemed not to get an obvious benefit (28). In HR-positive, HER2-negative, axillary node-negative breast cancer women with the midrange of RS, adjuvant chemoendocrine therapy and endocrine therapy appeared to have similar efficacy, but some chemotherapy benefits were present in a portion of patients aged ≤50 years (29). In the current study, however, no significant benefits were observed in high-risk patients with and without chemotherapy who were stratified by RecurIndex assay, which may be affected by various factors, such as small sample size, ethnic differences (30), different multigene assays and study populations, and require prospective validation in larger cohorts.

The intrinsic subtypes of breast cancer like luminal A, luminal B and HER2-enriched have been widely studied using microarray-based gene expression profiling, offering precise information for the prediction of recurrence risk in EBC (31). RecurIndex assay was developed with the aim to predict the risk of local and distant recurrences in Chinese EBC patients, and had been validated across diverse cohorts (11, 23, 24, 32). In the current study, we found that RecurIndex assay performed well in distinguishing low and high-risk patients with HR-positive and HER2-negative EBC. By contrast, the difference of 7-year DRFS rate was inapparent between luminal A and B patients assessed by IHC staining although the presence of superior 7-year RFS and LRFS rates in luminal A subtype, indirectly demonstrating the clinical utility of RecurIndex assay in a practical setting. Additionally, this study with large sample size was performed at 9 medical centers from China, highlighting the universality of our findings. Nevertheless, our study had several notable limitations. First, some important variables like menopausal status were missing due to the retrospective nature, thus we used an approximate menopausal status defined by an age cutoff at 50 years to assess the benefits of adjuvant chemotherapy (26). Second, significant differences were not shown between high-risk patients with and without adjuvant chemotherapy, which may result from the small number of patients not receiving adjuvant chemotherapy in high-risk cohort. In the future, we will conduct prospective studies with larger sample size to further confirm our findings.

In conclusion, HR-positive and HER2-negative EBC patients at low risk stratified by RecurIndex assay may be exempt from adjuvant chemotherapy to avoid treatment-related toxicities, while for high-risk patients, whether adjuvant chemotherapy may derive survival benefits still needs larger cohorts to verify.

## Data availability statement

The original contributions presented in the study are included in the article/**Supplementary Material**, further inquiries can be directed to the corresponding author/s.

## Ethics statement

The studies involving human participants were reviewed and approved by the Institutional Review Board of The Fourth Hospital of Hebei Medical University (approval No.: QYFYKYLL801311920). The patients/participants provided their written informed consent to participate in this study.

## Author contributions

HaW: Conceptualization, writing-original draft preparation, writing-editing and supervision; LM: Conceptualization, writing-reviewing and editing, supervision; YZ, OW, ZW, XX, XZ, JZ, QL and YR: Clinical sample collection, methodology and investigation; HuW, FD and SC: Data curation and formal analysis. All authors contributed to the article and approved the submitted version.

## Supplementary material

Supplementary Figure 1 Survival outcomes in low-risk patients with and without adjuvant chemotherapy. The 7-year recurrence-free survival rate **(A)** and distant recurrence-free survival rate **(B)** in patients aged 50 years or younger; The 7-year recurrence-free survival rate **(C)**, distant recurrence-free survival rate **(D)** in patients aged over 50 years; The 7-year recurrence-free survival rate **(E)**, distant recurrence-free survival rate **(F)** in patients receiving endocrine therapy.
