# Supplementary material for: RecurIndex assay as an aid for adjuvant chemotherapy decisions in HR-positive HER2-negative breast cancer patients
Source: Front Oncol. 2022 Dec 7;12:896431. doi: 10.3389/fonc.2022.896431 (PMC9769189; doi:10.3389/fonc.2022.896431)
Supplement: Supplementary file 1 [file Table_1.pdf]

Table S1. Baseline characteristics of 445 patients with pT1-2N0M0 Luminal-type breast cancer, n (%)

Characteristics	IHC assay		*p*
	Lum A (n=347)	Lum B (n=98)	
Median age (range)	51 (28-75)	49 (26-70)	0.082
Tumor stage			0.001
T1	254 (73.2)	55 (56.1)	
T2	93 (26.8)	43 (43.9)	
Histological grade			<0.001
I	48 (13.8)	0 (0.0)	
II	299 (86.2)	0 (0.0)	
III	0 (0.0)	98 (100.0)	
Lymphovascular invasion			0.026
Yes	49 (14.1)	23 (23.5)	
No	298 (85.9)	75 (76.5)	
Local recurrence			0.004
Yes	6 (1.7)	8 (8.2)	
No	341 (98.3)	90 (91.8)	
Distant recurrence			0.071
Yes	18 (5.2)	10 (10.2)	
No	329 (94.8)	88 (89.8)	
Adjuvant chemotherapy			<0.001
TC	96 (27.7)	40 (40.8)	
Anthracycline without taxane	34 (9.8)	11 (11.2)	
Anthracycline plus taxane	33 (9.5)	12 (12.2)	
CEF	14 (4.0)	13 (13.3)	
Others	15 (4.3)	3 (3.1)	
No chemotherapy	155 (44.7)	19 (19.4)	
Adjuvant endocrine therapy			0.029
Tam/TOR	174 (50.1)	58 (59.2)	
AI	126 (36.3)	27 (27.6)	
OFS+Tam/TOR	4 (1.2)	2 (2.0)	
OFS+AI	3 (0.9)	0 (0.0)	
Others	22 (6.3)	3 (3.1)	
None reported	9 (2.6)	0 (0.0)	
No endocrine therapy	9 (2.6)	8 (8.2)	

Abbreviations: TC, taxane and cyclophosphamide; CEF, cyclophosphamide/epirubicin/fluorouracil; OFS, ovarian function suppression; Tam, tamoxifen; TOR, toremifene; AI, aromatase inhibitor.
