# Supplementary figures and images for: RecurIndex assay as an aid for adjuvant chemotherapy decisions in HR-positive HER2-negative breast cancer patients
Source: Front Oncol. 2022 Dec 7;12:896431. doi: 10.3389/fonc.2022.896431 (PMC9769189; doi:10.3389/fonc.2022.896431)

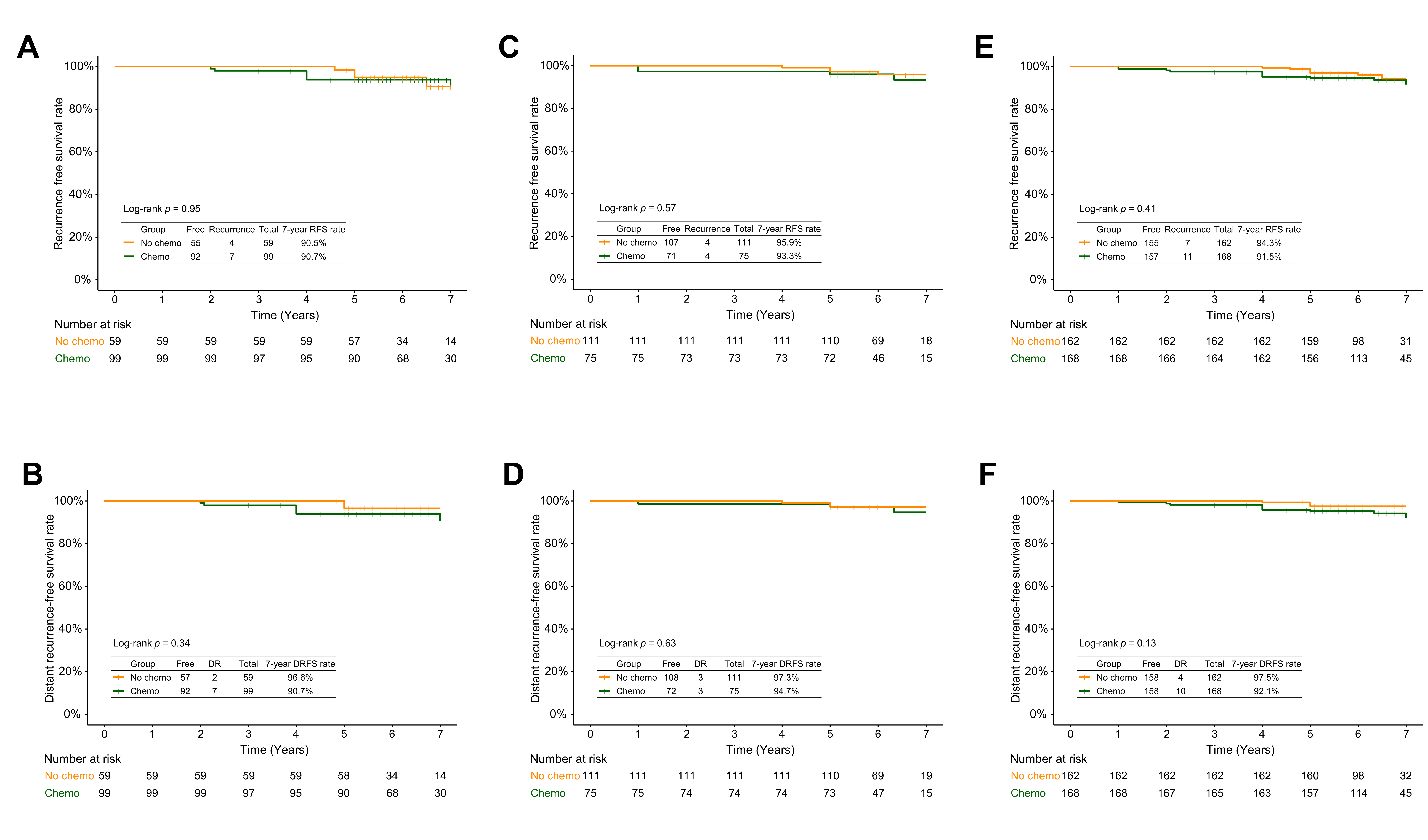

Supplement: Supplementary Figure 1 — Survival outcomes in low-risk patients with and without adjuvant chemotherapy. The 7-year recurrence-free survival rate (A) and distant recurrence-free survival rate (B) in patients aged 50 years or younger; The 7-year recurrence-free survival rate (C), distant recurrence-free survival rate (D) in patients aged over 50 years; The 7-year recurrence-free survival rate (E), distant recurrence-free survival rate (F) in patients receiving endocrine therapy. [file Image_1.tif]
